# Identification of microRNAs associated with the exogenous spermidine-mediated improvement of high-temperature tolerance in cucumber seedlings (*Cucumis sativus* L.)

**DOI:** 10.1186/s12864-018-4678-x

**Published:** 2018-04-24

**Authors:** Ying Wang, Shirong Guo, Lei Wang, Liwei Wang, Xueying He, Sheng Shu, Jin Sun, Na Lu

**Affiliations:** 1 College of Horticulture, Nanjing Agricultural University, Nanjing, 210095 China; 2 Nanjing Agricultural University (Suqian) Academy of Protected Horticulture, Suqian, 223800 Jiangsu China; 3 Center for Environment, Health and Field Sciences, Chiba University, Kashiwa-no-ha 6-2-1, Kashiwa, Chiba, Japan

**Keywords:** MicroRNA, Target gene, High-throughput sequencing, High-temperature stress, Spermidine; cucumber

## Abstract

**Background:**

High-temperature stress inhibited the growth of cucumber seedlings. Foliar spraying of 1.0 mmol·L^− 1^ exogenous spermidine (Spd) to the sensitive cucumber cultivar ‘Jinchun No. 2’ grown at high-temperature (42 °C/32 °C) in an artificial climate box improved the high-temperature tolerance. Although there have been many reports on the response of microRNAs (miRNAs) to high-temperature stress, the mechanism by which exogenous Spd may mitigate the damage of high-temperature stress through miRNA-mediated regulation has not been studied.

**Results:**

To elucidate the regulation of miRNAs in response to exogenous Spd-mediated improvement of high-temperature tolerance, four small RNA libraries were constructed from cucumber leaves and sequenced: untreated-control (CW), Spd-treated (CS), high-temperature stress (HW), and Spd-treated and high-temperature stress (HS). As a result, 107 known miRNAs and 79 novel miRNAs were identified. Eight common differentially expressed miRNAs (miR156d-3p, miR170-5p, miR2275-5p, miR394a, miR479b, miR5077, miR5222 and miR6475) were observed in CS/CW, HW/CW, HS/CW and HS/HW comparison pairs, which were the first set of miRNAs that responded to not only high-temperature stress but also exogenous Spd in cucumber seedlings. Five of the eight miRNAs were predicted to target 107 potential genes. Gene function and pathway analyses highlighted the integral role that these miRNAs and target genes probably play in the improvement of the high-temperature tolerance of cucumber seedlings through exogenous Spd application.

**Conclusions:**

Our study identified the first set of miRNAs associated with the exogenous Spd-mediated improvement of high-temperature tolerance in cucumber seedlings. The results could help to promote further studies on the complex molecular mechanisms underlying high-temperature tolerance in cucumber and provide a theoretical basis for the high-quality and efficient cultivation of cucumber with high-temperature resistance.

**Electronic supplementary material:**

The online version of this article (10.1186/s12864-018-4678-x) contains supplementary material, which is available to authorized users.

## Background

Cucumber (*Cucumis sativus* L.), an important vegetable crop worldwide, is also one of the main vegetable species grown in protected facilities. In many parts of China during the spring-summer and summer-autumn periods, the daytime air temperature in solar greenhouses and plastic greenhouses is often higher than the suitable temperature range for cucumber (25–30 °C) [1]. High-temperature stress seriously affects the normal growth of cucumber plants and can even lead to decreased yield and quality. This has been one of the primary factors limiting the summer-autumn protected cultivation of cucumber [2]. So far, few studies have focused on improving heat resistance through traditional breeding methods, and successful transgenic cases are also rare. In addition to genetic modification, pre-treatment with exogenous growth regulators can reduce damage from a variety of environmental stresses and could be an effective method to improve crop high-temperature tolerance [3, 4].

In recent years, studies have found that polyamines are closely related to plant stress resistance [5, 6]. Plants under stress will accumulate a large number of polyamines, such as spermine, putrescine and Spd. Polyamines not only increase the resistance of plants to salt stress [7], water stress [8], and mineral nutrient deficit [9], they also increase the plant’s ability to resist high temperatures [10]. Exogenous spermidine treatment can significantly change the content of endogenous polyamines [11] and effectively alleviate the inhibition of the growth of cucumber plants under high-temperature stress. High-temperature stress can cause significant changes in the expression of some proteins. Spraying exogenous Spd on the leaves can inhibit or promote the expression of some proteins and improve photosynthesis, energy and material metabolism, reactive oxygen species clearance and the biosynthesis of proteins and nucleic acids, which can promote the stress tolerance and improve the adaptability of plants to high temperature [12–14].

Plant miRNAs are endogenous non-coding single-stranded small RNAs with approximately 21 nucleotides (nt), which can control the expression of genes by cleaving target mRNAs or inhibiting the translation of proteins [15, 16]. They are first transcribed as stem-loop primary miRNAs by RNA polymerase II, then cleaved in the nucleus into mature miRNAs by Dicer-like 1 [17]. miRNAs widely control the growth of plants [18–20], organ formation [21–23], signal transduction pathways [24–26] and stresses response [27–29]. High-temperature can alter the expression of miRNAs. These miRNAs play an important role in the plant adaptation to high-temperature stress and restoring cell homeostasis [30]. The miRNA-mediated regulatory network in plants that responds to high-temperature stress is complex. Previous studies have identified a series of known and novel miRNAs in response to high-temperature stress in a variety of plants, such as *Brassica rapa* [31], *Arabidopsis* [32], *Oryza sativa* [33] and cucumber [34]. These results suggested that miRNA-mediated gene regulatory pathways may play an important role in plant adaptation to high-temperature.

Given the role of Spd in the response to high-temperature stress, we speculated that Spd might regulate target genes through miRNA-mediated regulation and enhance the high-temperature tolerance of cucumber seedlings to alleviate the damage to cucumber seedlings under high temperature. Although there have been many reports on the response of miRNAs to high-temperature stress, the mechanism by which exogenous Spd may mitigate the damage of high-temperature stress through miRNA-mediated regulation has not been studied. In our study, high-throughput sequencing technology was used to explore the miRNA-mediated regulation response to high-temperature and exogenous Spd, and the molecular mechanism by which exogenous Spd improves cucumber high-temperature tolerance was described based on the expression of miRNAs and their target genes. Our results provide useful information for identifying cucumber miRNAs that respond to exogenous Spd-mediated improvement of high-temperature tolerance, which may contribute to further studies of the complex molecular mechanisms of cucumber high-temperature tolerance and provide a theoretical basis for the high-quality and high-yield cultivation of cucumber that is resistant to high temperature.

## Results

### Effects of exogenous spermidine on the growth of cucumber seedlings under high-temperature stress

**Fig. 1:**
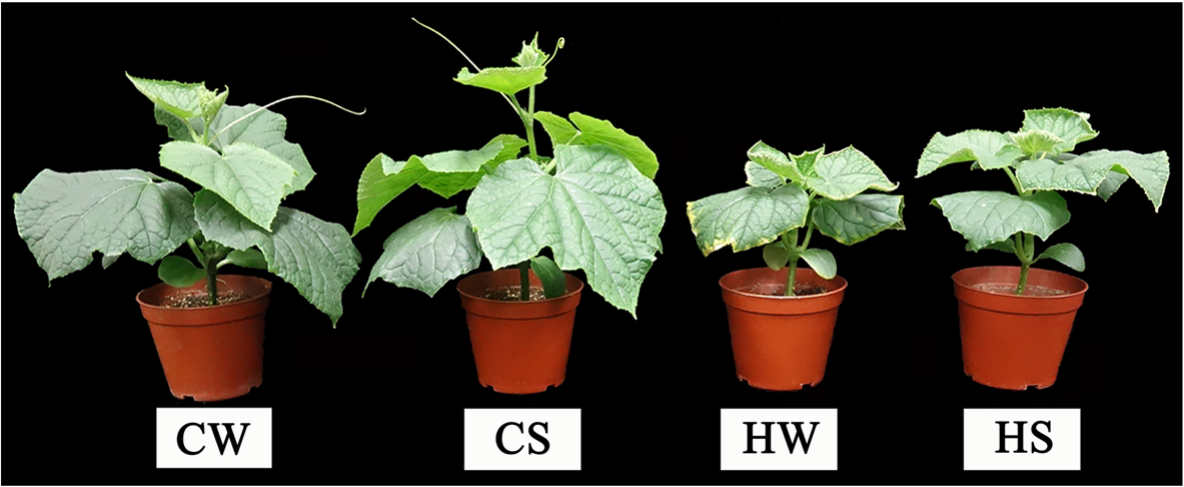
Effect of exogenous spermidine on shape of cucumber seedlings under high-temperature stress. (1) CW: Cucumber seedlings were sprayed with deionized water and maintained at 28 ± 1 °C/18 ± 1 °C; (2) CS: Cucumber seedlings were sprayed with 1 mmol·L^− 1^ Spd and maintained at 28 ± 1 °C/18 ± 1 °C; (3) HW: Cucumber seedlings were sprayed with deionized water and maintained at 42 ± 1 °C/32 ± 1 °C; (4) HS: Cucumber seedlings were sprayed with 1 mmol·L^− 1^ Spd and maintained at 42 ± 1 °C/32 ± 1 °C

As shown in Fig. 1, under the high-temperature stress condition, the cucumber seedlings were thin, and the size of the leaves was small. Spd spraying could effectively relieve the growth inhibition induced by high-temperature stress. Under high-temperature stress, the plant height, stem diameter, leaf area, fresh weight and dry weight of the cucumber seedling shoots decreased by 27.93%, 8.77%, 35.39%, 21.08% and 18.80%, respectively (Table 1). After spraying exogenous Spd, the plant height, stem diameter, leaf area and shoot fresh weight increased by 17.10%, 8.50%, 28.68% and 14.62%, respectively, compared to seedlings under high-temperature stress. However, there was no significant effect on dry weight. Foliar application of exogenous spermidine can therefore effectively relieve the inhibition of cucumber seedling growth under high-temperature stress.

**Table 1:** Effect of exogenous spermidine on growth of cucumber seedlings under high-temperature stress

Treatments	Plant height (cm·plant^− 1^)	Stem diameter (mm·plant^− 1^)	Leaf area (cm^2^·plant^− 1^)	Shoot	
				Fresh weight (g·plant^−1^)	Dry weight (g·plant^− 1^)
CW	11.60 ± 0.32a	5.93 ± 0.16a	82.23 ± 1.94b	14.99 ± 0.61b	1.17 ± 0.04ab
CS	12.49 ± 0.27a	6.31 ± 0.19a	91.99 ± 2.47a	16.58 ± 0.50a	1.30 ± 0.05a
HW	8.36 ± 0.129c	5.41 ± 0.10b	53.13 ± 1.81d	11.83 ± 0.40c	0.95 ± 0.04c
HS	9.79 ± 0.37b	5.87 ± 0.13a	68.37 ± 2.64c	13.56 ± 0.54b	1.08 ± 0.06bc

Different letters indicate significant difference at 0.05 level. The same as follows

### Small RNA libraries sequence analysis

To explore the expression patterns of small RNAs (sRNAs) in response to high-temperature and exogenous Spd, four RNA libraries were constructed: CW, CS, HW, and HS. These four libraries generated 11,255,470, 12,493,521, 11,296,147, and 11,253,964 raw reads, respectively (Table 2). Of these raw reads, 10,865,804 (96.62%), 12,215,270 (97.87%), 10,854,344 (96.19%) and 10,884,228 (96.82%), respectively, remained after the contaminant and low-quality sequences were removed. The total number of sRNA reads was 44,819,646, while the total number of unique reads was 3,882,844, and the average number of reads for each sequence was 12.

**Table 2:** Summary of cleaning data from CW, CS, HW and HS sRNA libraries

Type	CW		CS		HW		HS	
	Count	Percent (%)	Count	Percent (%)	Count	Percent (%)	Count	Percent (%)
Total reads	11,255,470		12,493,521		11,296,147		11,253,964	
High quality	11,245,483	100	12,481,499	100	11,284,130	100	11,242,205	100
3’adapter null	36,410	0.32	42,423	0.34	48,705	0.43	47,563	0.42
Insert null	863	0.01	630	0.01	2094	0.02	1018	0.01
5’adapter contaminants	9749	0.09	6490	0.05	14,824	0.13	11,375	0.10
Smaller than 18 nt	332,565	2.96	216,543	1.73	364,094	3.23	297,956	2.65
Poly(A)	92	0.00	143	0.00	69	0.00	65	0.00
Clean reads	10,865,804	96.62	12,215,270	97.87	10,854,344	96.19	10,884,228	96.82

**Fig. 2:**
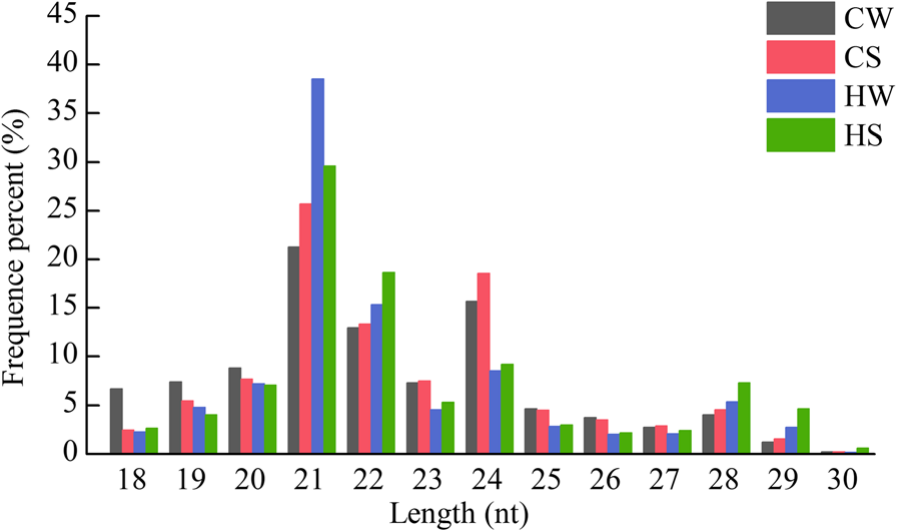
Length distribution of small RNAs in CW, CS, HW and HS libraries. Y-axis represents percentages of sRNAs identified in the study; X-axis represents the length of sRNAs. Four libraries are shown by different colors

Quality statistics and length statistics were done for the filtered clean data. As shown in Additional file 1: Figure S1, the quality of bases in CW, CS, HW and HS libraries met the sequencing requirements, which ranged from 0 to 41. Figure 2 shows the length distribution of sRNAs in cucumber seedlings from the four libraries. The sRNA length was typically 19 nt to 24 nt. The most common length was 21 nt, accounting for 21.28%, 25.70%, 38.51% and 29.61% of the sRNAs in the CW, CS, HW and HS libraries, respectively.

### Small RNA classification and annotation

To classify the sRNAs, all clean reads in the four libraries were compared to the Cucumber Genomics Database, the miRBase 21.0 database, GenBank and Rfam databases. The clean sequences were aligned with the genomic data, resulting in 5,802,084 reads (53.4%) and 682,125 types (65.58%) of sequences in the CW library; 7,829,913 reads (64.1%) and 869,558 types (67.68%) of sequences in the CS library; 7,339,246 reads (67.62%) and 435,395 types (59.69%) of sequences in the HW library; and 6,895,911 reads (63.36%) and 496,189 types (59.90%) of sequences in the HS library. Finally, the sRNAs were divided into tRNA, rRNA, small nuclear RNA (snRNA), small nucleolar RNA (snoRNA), repeat sequences and miRNA according to the matching results with each database.

For known miRNAs, the CW library matched 6432 types and 1,575,878 reads of sequences, accounting for 0.62% and 14.50%, respectively. The CS library matched 7270 types and 2,148,690 reads of sequences, accounting for 0.57% and 17.59%, respectively. The HW library matched 7195 types and 3,722,103 reads of sequences, accounting for 0.99% and 34.29%, respectively. The HS library matched 7145 types and 2,612,864 reads of sequences, accounting for 0.86% and 24.01%, respectively (Table 3).

**Table 3:** Summary and classification of small RNAs in CW, CS, HW and HS libraries

Category	CW		CS		HW		HS	
	Unique sRNA (%)	Total sRNA (%)	Unique sRNA (%)	Total sRNA (%)	Unique sRNA (%)	Total sRNA (%)	Unique sRNA (%)	Total sRNA (%)
exon antisense	7067 (0.68)	17,282 (0.16)	14,310 (1.11)	31,030 (0.25)	5331 (0.73)	11,341 (0.10)	5901 (0.71)	15,843 (0.15)
exon sense	26,976 (2.59)	43,069 (0.40)	45,302 (3.53)	77,587 (0.64)	26,834 (3.68)	44,971 (0.41)	31,928 (3.85)	52,498 (0.48)
intron antisense	15,574 (1.50)	45,849 (0.42)	20,490 (1.59)	66,541 (0.54)	11,238 (1.54)	46,639 (0.43)	12,190 (1.47)	39,875 (0.37)
intron sense	24,802 (2.38)	78,844 (0.73)	31,903 (2.48)	105,302 (0.86)	18,472 (2.53)	61,713 (0.57)	20,467 (2.47)	61,559 (0.57)
rRNA	61,369 (5.90)	2,184,736 (20.11)	68,166 (5.31)	2,649,461 (21.69)	48,059 (6.59)	1,389,649 (12.80)	52,602 (6.35)	1,527,028 (14.03)
snRNA	2259 (0.22)	23,346 (0.21)	4234 (0.33)	48,054 (0.39)	2408 (0.33)	30,015 (0.28)	3316 (0.40)	46,895 (0.43)
snoRNA	2652 (0.25)	24,435 (0.22)	3388 (0.26)	32,840 (0.27)	2625 (0.36)	22,373 (0.21)	2958 (0.36)	23,719 (0.22)
tRNA	6487 (0.62)	141,212 (1.30)	8732 (0.68)	222,821 (1.82)	7140 (0.98)	156,167 (1.44)	9152 (1.10)	237,356 (2.18)
miRNA	6432 (0.62)	1,575,878 (14.50)	7270 (0.57)	2,148,690 (17.59)	7195 (0.99)	3,722,103 (34.29)	7145 (0.86)	2,612,864 (24.01)
unannotated	886,575 (85.23)	6,731,153 (61.95)	1,081,103 (84.14)	6,832,944 (55.94)	600,151 (82.27)	5,369,373 (49.47)	682,641 (82.41)	6,266,591 (57.57)
total	1,040,193	10,865,804	1,284,898	12,215,270	729,453	10,854,344	828,300	10,884,228

A total of 107 known miRNAs belonging to 85 miRNA families were identified in our study after removed the miRNAs that expressed only in one library (Additional file 2: Table S1). Among them, 93 known miRNAs were expressed in the CW library, and the average number of reads was 17,755; 93 known miRNAs were expressed in the CS library, and the average number of reads was 24,171; 84 known miRNAs were expressed in the HW library, and the average number of reads was 44,725; and 79 known miRNAs were expressed in the HS library, and the average number of reads was 33,153. There were 61 known miRNAs belonging to 53 miRNA families that were shared by all four libraries. Among them, the largest family was the miR156/157 family, with 4 members, followed by the miR165/166 and miR319 families, with 3 members. In addition, the expression levels can be deduced according to the number of miRNA reads in cucumber seedlings. The level of expression differed significantly among different family members. For example, miR166a-3p had more than one million reads in all four libraries, indicating that it was expressed at a very high level. Similarly, miR396f, miR7767-5p, miR166u and miR159a were also highly expressed. However, fewer than 30 reads for miR393a-3p and miR829-3p were found in the four libraries, meaning that they were expressed at a low level. Furthermore, the expression of members in the same family usually considered to be similar may also be very different. For example, in the miR156/157 family, miR157a-5p was expressed at a high level, while the expression of miR157d-3p was low. The large differences in expression between different members of the same family suggested that miRNAs were precisely expressed under certain conditions.

After the first round of annotation, a large number of reads from the four libraries remained (Table 3). These sRNA sequences, which were not annotated with any known sRNAs, were used to predict novel candidate miRNAs. A total of 79 novel miRNAs were predicted in the four libraries after removed the miRNAs that expressed only in one library (Additional file 3: Table S2). Among them, there were 44 novel miRNAs in CW library; there were 53 novel miRNAs in CW library; there were 52 novel miRNAs in CW library; there were 58 novel miRNAs in CW library. The expression levels of these novel miRNAs in the four libraries were different, but in general, the expression of the novel miRNAs and the number of family members were much lower than that of known miRNAs.

### Identification of miRNAs responsive to high-temperature and exogenous spermidine

To find miRNAs that responded to exogenous Spd to alleviate high-temperature stress and identify their expression patterns, we divided these four libraries into four comparison pairs: CS/CW, HW/CW, HS/CW and HS/HW. Four sets of differentially expressed miRNAs were obtained by comparing miRNA expression levels between the two compared libraries in each comparison pair. Among them, 29 differentially expressed known miRNAs and 36 differentially expressed novel miRNAs were obtained in the CS/CW comparison pair; 73 differentially expressed known miRNAs and 50 differentially expressed novel miRNAs were obtained in the HW/CW comparison pair; 73 differentially expressed known miRNAs and 50 differentially expressed novel miRNAs were obtained in the HS/CW comparison pair; and 32 differentially expressed known miRNAs and 26 differentially expressed novel miRNAs were obtained in the HS/HW comparison pair.

**Fig. 3:**
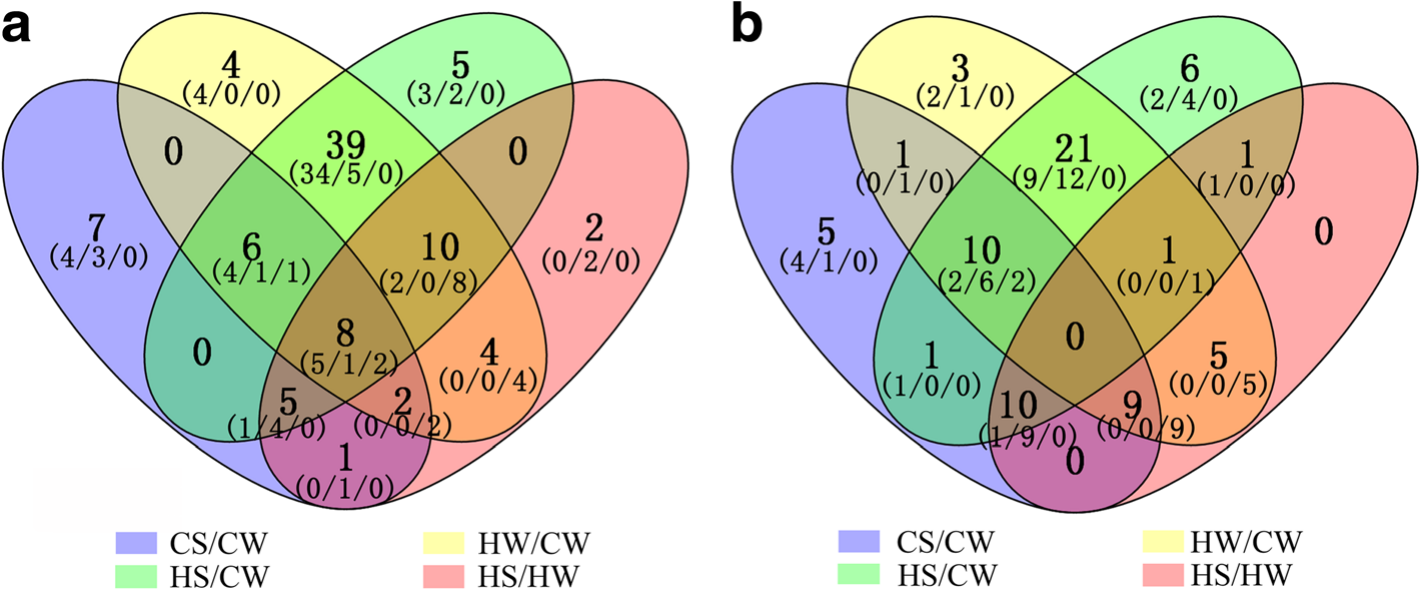
Venn diagrams for analysis of miRNAs differentially expressed in four comparison pairs. (**a**) Venn diagrams for analysis of know miRNAs differentially expressed in the CS/CW, HW/CW, HS/CW and HS/HW comparison pairs from cucumber seedlings. (**b**) Venn diagrams for analysis of novel miRNAs differentially expressed in the CS/CW, HW/CW, HS/CW and HS/HW comparison pairs from cucumber seedlings. There are three numbers in parentheses connected by a slash. The first one indicated the number of miRNAs whose expression were down-regulated, the second one indicated the number of miRNAs whose expression were up-regulated, and the third one indicated the number of miRNAs whose expression patterns were inconsistent in different comparison pairs

A Venn diagram visually showed the numbers of common and specific miRNAs expressed differentially among the four comparison pairs (Fig. 3). It should be noted that the 63 known miRNAs and 32 novel miRNAs that were co-expressed differentially in the HW/CW and HS/CW comparison pairs may respond to high-temperature stress (Additional file 4: Table S3). The 13 known miRNAs and 10 novel miRNAs that were co-expressed differentially in the CS/CW, HS/CW and HS/HW comparison pairs may respond to exogenous Spd (Additional file 5: Table S4). In general, most known miRNAs were down-regulated in response to high-temperature stress or Spd, whereas novel miRNAs were mostly up-regulated. Interestingly, some miRNAs had different patterns of expression in different comparison pairs, but many miRNAs had similar expression patterns. For example, both miR5077 and miR6475 were down-regulated in response to high-temperature stress. They were down-regulated in response to Spd at normal temperatures, but up-regulated in response to Spd at high-temperatures, suggesting that their responsive mechanisms to Spd under high-temperature and normal temperature were not exactly the same (Fig. 3). In addition, the 8 known miRNAs that were co-expressed differentially in all four comparison pairs may respond to both high-temperature stress and exogenous Spd and are likely to play important roles in the improvement of high-temperature tolerance in cucumber by Spd. To be prudent,we used two-way ANOVA to validate the 8 miRNAs selected in our study. The specific results were shown in Additional file 6: Table S5. As a result, there were indeed interaction between high-temperature and Spd in the 8 miRNAs. All the F-values were greater than 0.05 and *P*-values were less than 0.05, indicating significant differences. In other words, the selection of the 8 miRNAs that we really focused on in this study were credible (Additional file 6: Table S5).

Of these eight miRNAs, five miRNAs (miR156d-3p, miR2275b-5p, miR394a, miR479b and miR5222) were down-regulated in the four comparison pairs, two miRNAs (miR5077 and miR6475) were up-regulated only in the HS/HW comparison pair and were down-regulated in the other three comparison pairs, and one miRNA (miR170-5p) was down-regulated in the HS/HW comparison pair and up-regulated in the other three comparison pairs. Moreover, miR6475 had the most up-regulated expression in the HS/HW comparison pair, with a fold-change of 6.3084, while miR156d-3p had the most down-regulated expression in the HS/CW comparison pair, with a fold-change of − 10.6731 (Table 4).

**Table 4:** List of miRNAs significantly expressed differentially in the CS/CW, HW/CW, HS/CW and HS/HW comparison pairs

miRNA	CS/CW			HW/CW			HS/CW			HS/HW		
	Fold-change Log_2_(CS/CW) normalized	*P*-value	Significance	Fold-change Log_2_(HW/CW) normalized	*P*-value	Significance	Fold-change Log_2_(HS/CW) normalized	*P*-value	Significance	Fold-change Log_2_(HS/HW) normalized	*P*-value	Significance
miR156d-3p	−10.4348	0.0000	**	−1.3993	0.0000	**	− 10.6731	0.0000	**	− 9.2738	0.0000	**
miR170-5p	3.1855	0.0000	**	3.7780	0.0000	**	2.6636	0.0000	**	4.8229	0.0001	**
miR2275b-5p	− 6.2535	0.0000	**	− 1.2379	0.0002	**	− 6.4918	0.0000	**	− 5.2539	0.0000	**
miR394a	− 5.2535	0.0000	**	− 1.5333	0.0009	**	− 5.4918	0.0000	**	− 3.9584	0.0009	**
miR479b	− 1.8756	0.0000	**	− 2.1893	0.0000	**	− 10.2119	0.0000	**	− 8.0225	0.0000	**
miR5077	− 2.7722	0.0000	**	− 4.4742	0.0000	**	− 1.7235	0.0000	**	2.7507	0.0000	**
miR5222	− 1.2117	0.0001	**	− 2.0505	0.0000	**	− 7.3568	0.0000	**	− 5.3063	0.0000	**
miR6475	− 1.7092	0.0000	**	− 8.3554	0.0000	**	− 2.0470	0.0000	**	6.3084	0.0000	**

**P-value < 0.01

### Genes and pathways involved in the high-temperature stress and exogenous spermidine response

In our study, we identified 8 important known miRNAs whose expression was significantly different in all four comparison pairs (CS/CW, HW/CW, HS/CW and HS/HW), which implied they were likely to respond to both high-temperature stress and exogenous Spd. In other words, these miRNAs may play important roles in the process of Spd-mediated improvement of heat tolerance in cucumber. Finally, 107 genes were predicted to be targeted by 5 of these known miRNAs, and no gene were found to be targeted by the other 3 miRNAs (miR170-5p, miR2275-5p and miR5222). The number of genes targeted by each miRNA ranged from 1 to 96, with miR6475 having the highest number of predicted target genes (Additional file 7: Table S6).

**Fig. 4:**
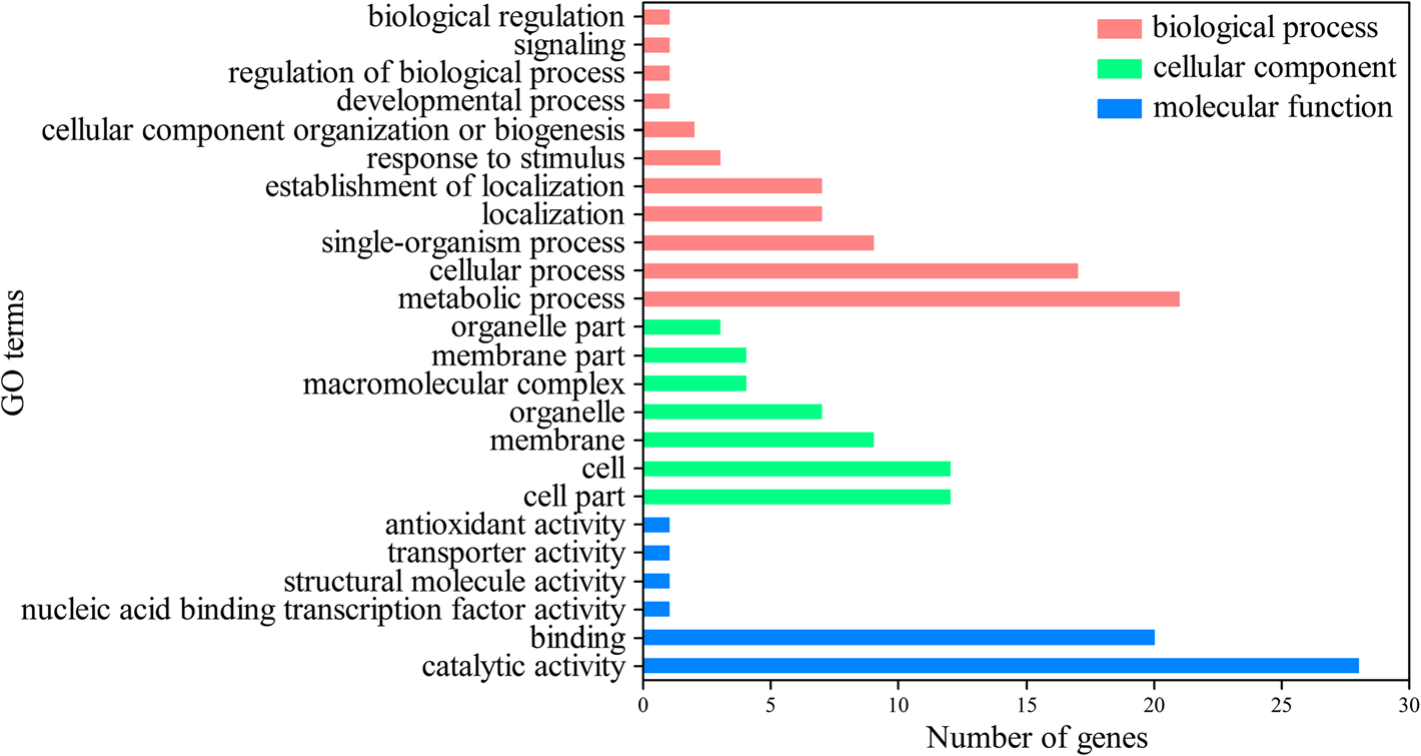
Gene ontology classification of gene candidates targeted by miRNAs responding to high-temperature and spermidine

These genes were then subjected to GO (Gene Ontology) annotation classification and KEGG (Kyoto Encyclopedia of Genes and Genome) metabolic pathway analysis. Of all the target gene candidates, only 45 genes targeted by miR394a, miR479b, miR5077 and miR6475 were successfully assigned to the corresponding 24 GO terms respectively. The predominant subcategories in the biological processes were “metabolic process” (GO: 0044710) and “cellular process” (GO: 0009987). The main subcategories in the cellular components were “cell” (GO: 0005623) and “cell part” (GO: 0044464). The most abundant subcategories in the molecular functions were “catalytic activity” (GO: 0003824) and “binding” (GO: 0005488) (Fig. 4). The GO enrichment analysis showed that nine genes were significantly enriched in two of the GO terms (Table 5). These two GO terms were intracellular membrane-bound organelle (GO: 0043231) and nucleic acid binding (GO: 0003676). The protein products encoded by the nine target genes were Methanol dehydrogenase (precursor), Rac-like GTP-binding protein, Ferredoxin I, 3-beta-hydroxysteroid-delta-isomerase, MADS-box transcription factor 2, Outer envelope pore protein 37, Chromodomain-helicase-DNA-binding protein 1-like, 30S ribosomal protein S5 and Elongation factor EF-2. Interestingly, these nine target genes were only targeted by miR5077 and miR6475. Although gene candidates targeted by the other miRNAs (miR394a, miR479b) were involved in mapping Go terms, their *p*-values were greater than 0.05 which indicated there was no statistically significant.

**Table 5:** The significantly enriched GO terms of genes targeted by miRNAs responding to high-temperature and spermidine

Comparison pairs	GO term	Ontology	Class	*P*-value	Gene ID	Putative protein coding by target genes	miRNA name
HW/CW	GO:0003676	Molecular function	nucleic acid binding	0.0256	Csa3M305660	Chromodomain-helicase-DNA-binding protein 1-like	miR6475
					Csa4M166960	30S ribosomal protein S5	miR6475
					Csa5M172800	MADS-box transcription factor 2	miR6475
					Csa6M176410	Elongation factor EF-2	miR6475
	GO:0043231	Cellular component	intracellular membrane-bounded organelle	0.0195	Csa2M020910	Methanol dehydrogenase (Precursor)	miR6475
					Csa3M002670	Rac-like GTP-binding protein	miR6475
					Csa4M007050	3-beta-hydroxysteroid-delta-isomerase	miR5077
					Csa5M172800	MADS-box transcription factor 2	miR6475
					Csa6M410680	Outer envelope pore protein 37, chloroplastic	miR6475
HS/HW	GO:0043231	Cellular component	intracellular membrane-bounded organelle	0.0389	Csa2M020910	Methanol dehydrogenase (Precursor)	miR6475
					Csa3M002670	Rac-like GTP-binding protein	miR6475
					Csa3M222800	Ferredoxin I	miR6475
					Csa4M007050	3-beta-hydroxysteroid-delta-isomerase	miR5077
					Csa5M172800	MADS-box transcription factor 2	miR6475
					Csa6M410680	Outer envelope pore protein 37, chloroplastic	miR6475

The GO terms with a corrected *P*-value < 0.05 were defined as significantly enriched in target gene candidates

The target gene candidates mapping to KEGG pathways were consistent with those mapping to GO, with 83 KEGG pathways. Many KEGG pathways were significantly enriched in the four comparison pairs (Table 6). There were 2 pathways that were significantly enriched in the CS/CW comparison pair, which involved 3 target genes. There were 4 pathways that were enriched in the HW/CW comparison pair, which involved 6 target genes. There were 4 pathways that were enriched in the HS/CW comparison pair, which involved 6 target genes. And there were 5 pathways that were enriched in the HS/HW comparison pair, which involved 8 target genes. It was worth mentioning that, all the target genes were targeted by miR6475. Among them, the translational activator GCN1 and the cell differentiation protein rcd1 were significantly enriched in the RNA degradation pathway (ko03018) in all four comparison pairs. These metabolic pathways were involved in RNA metabolism (ko03018), ribosome biogenesis in eukaryotes (ko03008), secondary metabolism (ko00900, ko04977) and other metabolites. However, the *p*-values of the other pathways were greater than 0.05, indicating no statistically significant (Additional file 8: Table S7).

**Table 6:** The significantly enriched KEGG pathways of genes targeted by miRNAs responding to high-temperature and spermidine

Comparison pairs	Pathway ID	Pathways	*P*-value	Gene ID	Putative protein coding by target genes	miRNA name
CS/CW	ko00900	Terpenoid backbone biosynthesis	0.0173	Csa7M212680	UPF0505 protein C16orf62-like	miR6475
	ko03018	RNA degradation	0.0064	Csa2M079640	Translational activator GCN1	miR6475
	ko03018	RNA degradation	0.0064	Csa7M214190	Cell differentiation protein rcd1, putative	miR6475
HW/CW	ko00900	Terpenoid backbone biosynthesis	0.0007	Csa7M212680	UPF0505 protein C16orf62-like	miR6475
	ko03008	Ribosome biogenesis in eukaryotes	0.0007	Csa2M079640	Translational activator GCN1	miR6475
	ko03008	Ribosome biogenesis in eukaryotes	0.0007	Csa4M279830	Oligo ribonuclease	miR6475
	ko03008	Ribosome biogenesis in eukaryotes	0.0007	Csa3M686720	WGS project CAID00000000 data, contig chromosome 07	miR6475
	ko03018	RNA degradation	0.0010	Csa2M079640	Translational activator GCN1	miR6475
	ko03018	RNA degradation	0.0010	Csa7M214190	Cell differentiation protein rcd1, putative	miR6475
	ko04977	Vitamin digestion and absorption	0.0035	Csa5M603280	Nucleobase ascorbate transporter	miR6475
HS/CW	ko00900	Terpenoid backbone biosynthesis	0.0000	Csa7M212680	UPF0505 protein C16orf62-like	miR6475
	ko03008	Ribosome biogenesis in eukaryotes	0.0000	Csa2M079640	Translational activator GCN1	miR6475
	ko03008	Ribosome biogenesis in eukaryotes	0.0000	Csa3M686720	WGS project CAID00000000 data, contig chromosome 07	miR6475
	ko03008	Ribosome biogenesis in eukaryotes	0.0000	Csa4M279830	Oligo ribonuclease	miR6475
	ko03018	RNA degradation	0.0000	Csa7M214190	Cell differentiation protein rcd1, putative	miR6475
	ko03018	RNA degradation	0.0000	Csa2M079640	Translational activator GCN1	miR6475
	ko04977	Vitamin digestion and absorption	0.0062	Csa5M603280	Nucleobase ascorbate transporter	miR6475
HS/HW	ko00900	Terpenoid backbone biosynthesis	0.0054	Csa7M212680	UPF0505 protein C16orf62-like	miR6475
	ko03008	Ribosome biogenesis in eukaryotes	0.0000	Csa2M079640	Translational activator GCN1	miR6475
	ko03008	Ribosome biogenesis in eukaryotes	0.0000	Csa3M686720	WGS project CAID00000000 data, contig chromosome 07	miR6475
	ko03008	Ribosome biogenesis in eukaryotes	0.0000	Csa4M279830	Oligo ribonuclease	miR6475
	ko03018	RNA degradation	0.0000	Csa7M214190	Cell differentiation protein rcd1, putative	miR6475
	ko03018	RNA degradation	0.0000	Csa2M079640	Translational activator GCN1	miR6475
	ko04977	Vitamin digestion and absorption	0.0006	Csa5M603280	Nucleobase ascorbate transporter	miR6475
	ko05202	Transcriptional misregulation in cancer	0.0370	Csa5M180300	Dual specificity protein phosphatase, putative	miR6475
	ko05202	Transcriptional misregulation in cancer	0.0370	Csa7M325200	ATP-dependent RNA helicase	miR6475

The pathways with a corrected *P*-value < 0.05 were defined as significantly enriched in target gene candidates

qRT-PCR validation of the high-temperature-responsive and exogenous spermidine-responsive miRNAs and their potential target genes.

**Fig. 5:**
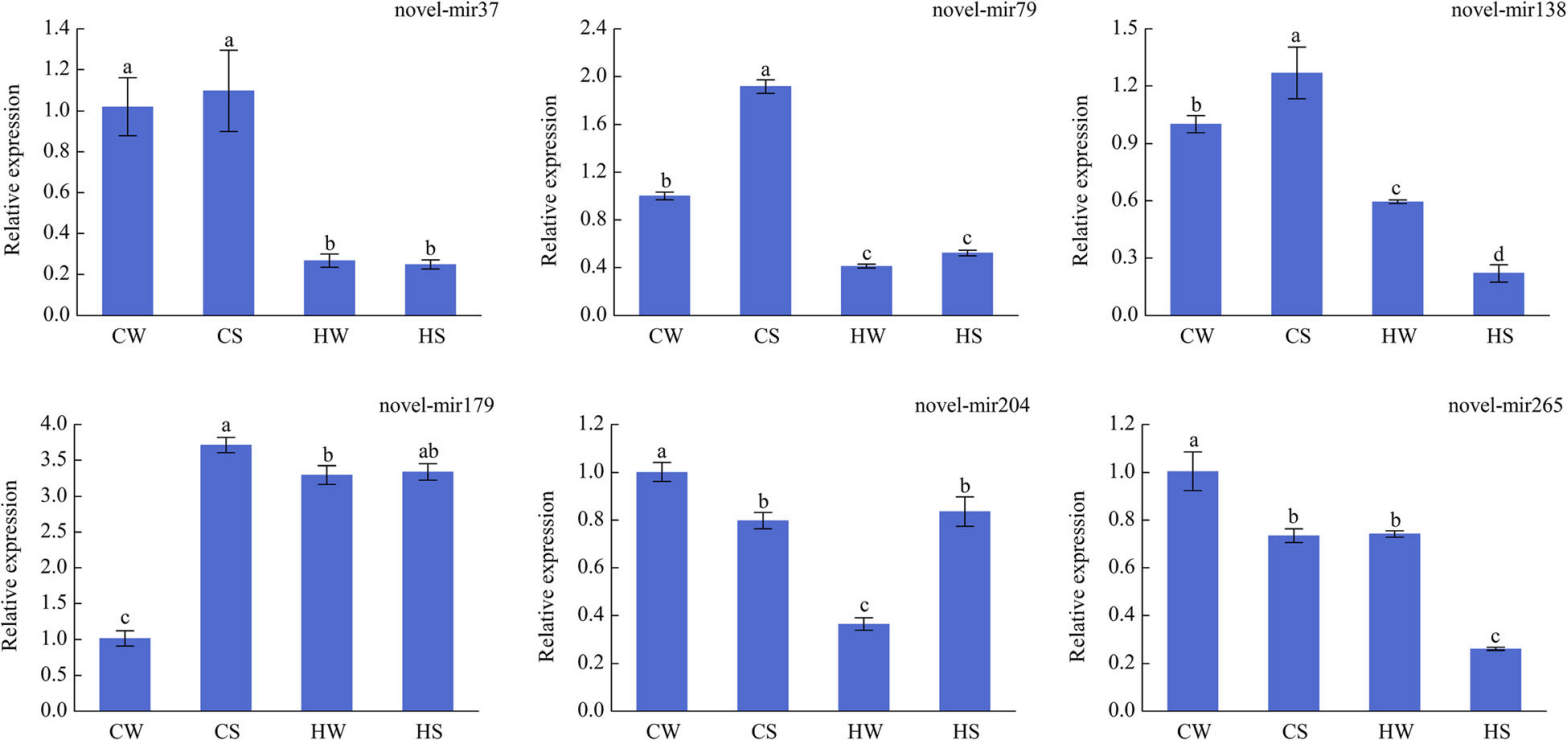
qRT-PCR analysis of novel miRNAs miRNA expression values shown are relative to the expression in CW sample, whose expression value was set to one. Error bars indicate ± SD calculated from three biological replicates. Values marked by different letters are significantly different with Student’s t-test (*p*-value < 0.05, *n* = 3)

**Fig. 6:**
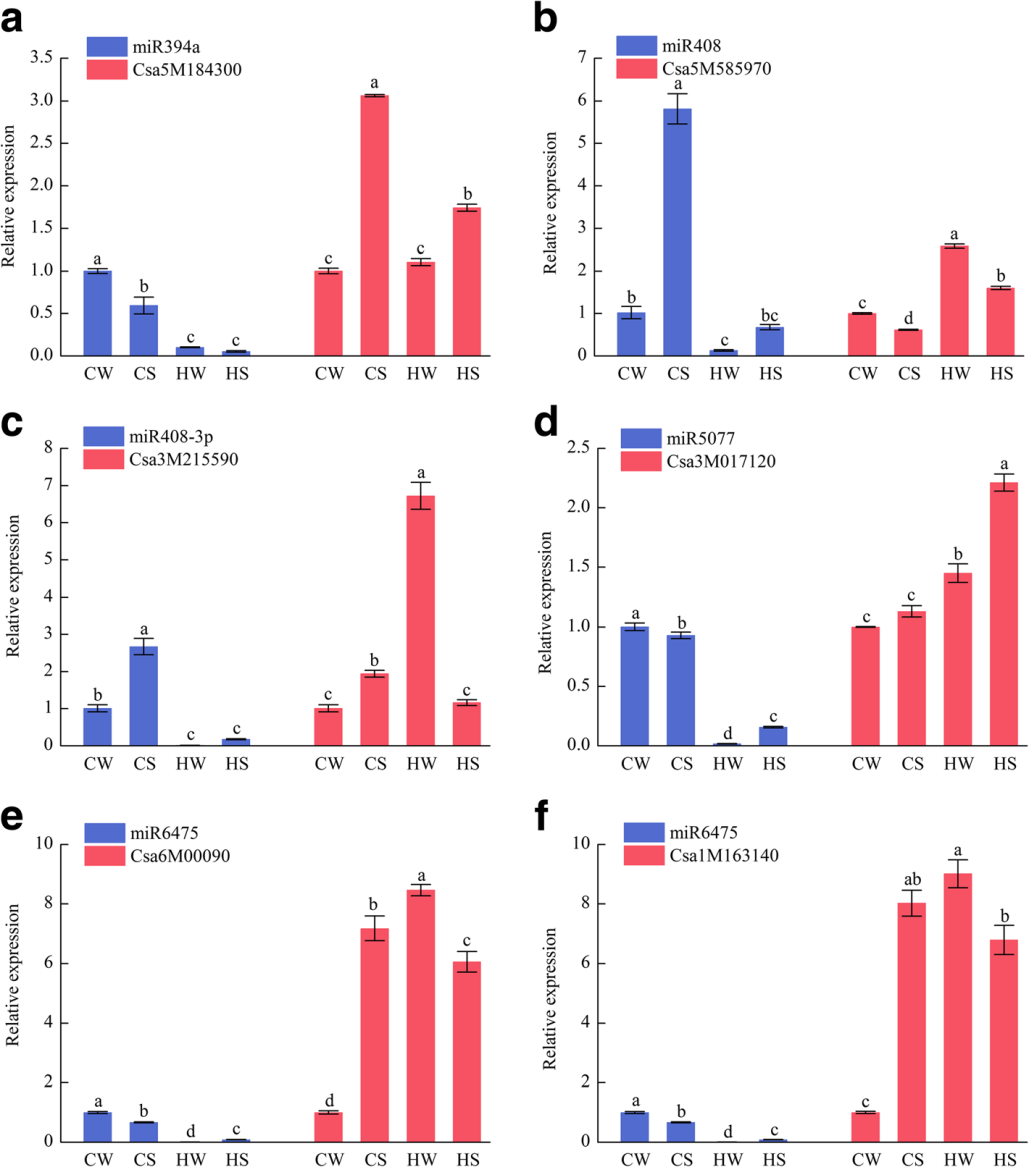
qRT-PCR analysis of several miRNA-targets. **a** Expression patterns of miR394a and its target gene S-adenosyl-L-methionine-dependent methyltransferase (Csa5M184300). **b** Expression patterns of miR408 and its target gene Peptide chain release factor (Csa5M585970). **c** Expression patterns of miR408-3p and its target gene Basic blue copper protein (Csa3M215590). **d** Expression patterns of miR5077 and its target gene Cyclophilin type peptidyl-prolyl cis-trans isomerase (Csa3M017120). **e** Expression patterns of miR6475 and its target gene SWR1-complex protein 5 (Csa6M00090). **f** Expression patterns of miR6475 and its target gene Ribosomal protein (Csa1M163140). Blue and red represent miRNAs and their corresponding target genes, respectively. miRNA expression values shown are relative to the expression in CW sample, whose expression value was set to one. Error bars indicate ± SD calculated from three biological replicates. Values marked by different letters are significantly different with Student’s t-test (*p*-value < 0.05, *n* = 3)

qRT-PCR is an authoritative standard for quantifying miRNAs because of its specificity and sensitivity. The six novel miRNAs associated with high-temperature and/or Spd were quantified by qRT-PCR (Fig. 5). Five known miRNAs and their corresponding six target genes quantified by qRT-PCR were shown in Fig. 6. The results showed that the differential expression of miRNAs by qRT-PCR was generally consistent with the sequencing results. For example, the expression of novel-mir37 was down-regulation in response to high-temperature, but did not change significantly after spraying Spd. And the expression of novel-mir265 was down-regulation in response to Spd. For known miRNAs, miR6475 was significantly down-regulated in the CS/CW, HW/CW, HS/CW, and up-regulated in HS/HW comparison pairs, consistent with the high-throughput sequencing results.

In addition, the expression patterns of six target genes for these five known miRNAs were validated to confirm the correlation between the differentially expressed miRNAs and their target genes. The six target genes were S-adenosyl-L-methionine-dependent methyltransferase, peptide chain release factor, basic blue copper protein, cyclophilin-like peptidyl-prolyl cis-trans isomerase domain, SWR1-complex protein 5 and ribosomal protein L1. The results revealed that miRNAs were inversely related to their target genes. For example, the expression of miR394a was down-regulated in the CS/CW, HW/CW and HS/CW comparison pairs, but its target genes were up-regulated; miR394a was down-regulated in the HS/HW comparison pair, while its target gene was up-regulated. Most notably, we identified two target genes for miR6475 whose expression was negatively correlated with that of miR6475, suggesting that one miRNA can indeed target multiple genes. The similar changes in the miRNA expression pattern identified by both qRT-PCR and high-throughput sequencing indicated that the high temperature-responsive and/or exogenous Spd-responsive miRNAs identified in this study were credible.

## Discussion

It is well known that the roles of miRNAs in the regulation of gene expression and in the plant response to biological and abiotic stresses are ubiquitous. In recent years, more and more reports have indicated that miRNA-mediated gene regulation plays an important role in the plant response to high-temperature stress. Cucumber is an important vegetable crop worldwide. High-temperature stress leads to low yield and bad quality in cucumber. Spraying exogenous Spd can effectively alleviate the injury to cucumber seedlings caused by high-temperature stress (Table 1; Fig. 1). To investigate the responsive mechanism of miRNAs to the Spd-mediated alleviation of damage to cucumber seedlings from high-temperature stress, this study attempted to identify miRNAs responsive to high temperature and exogenous Spd, which may play a role in the regulation process by regulating the differential expression of a series of genes under high-temperature stress.

### Expression of small RNAs in cucumber seedlings

High-temperature stress is one of the most important abiotic stress factors. It has been shown in many plants that high-temperature can change the expression of miRNAs, such as in French bean [35], radish [36] and wheat [37]. These miRNAs, play an important role in adapting to high-temperature stress and restoring cell homeostasis.

Plant miRNAs are generated by the precise cleavage of 21 nt sRNAs from the stem-loop secondary structure of the miRNA precursor [38]. In our study, 21 nt sRNAs were the most abundant in the CW, CS, HW and HS libraries, and their expression was significantly increased under high-temperature stress and markedly decreased after treatment with exogenous Spd in cucumber seedlings. This indicated that the 21 nt sRNAs were likely to be the most commonly expressed in response to high-temperature stress or exogenous Spd (Fig. 2).

The number and expression abundance of family members determined the diversity of cucumber miRNA families. In our study, the known miRNA families were shown to have higher expression abundance than the novel miRNA families (Additional files 2, 3: Table S1, S2), which was consistent with previous findings in cucumber [39].

### MiRNAs involved in the response to both high-temperature and exogenous spermidine

Our study identified eight known miRNAs that were likely to respond to both high-temperature and exogenous Spd (Table 4). Among these miRNAs, the highly conserved miR394a has been reported in many studies demonstrating that miR394 is involved in abiotic stress regulation. Li et al., (2011) found that miR394a was up-regulated in response to drought stress and down-regulated in response to salt stress in soybean by deep sequencing and qRT-PCR [40]. This indicated that miR394a is involved in a complex network that responds to multiple environmental stresses. In this study, miR394a was significantly down-regulated under high-temperature stress and sharply declined after exogenous Spd treatment, indicating that miR394a may respond to both high-temperature stress and exogenous Spd.

The up-regulation of miR156 can regulate plant morphology by regulating the transcription of squamosa promoter binding protein to help plants adapt to stresses [41, 42]. miR156 was suppressed by high-temperature stress [43]. In our study, miR156d-3p, which belongs to the miR156/157 family, was down-regulated in all four comparison pairs. However, another study has shown that, bra-miR156h and bra-miR156g were heat-induced [44]. This may be caused by the diversity of expression among the members of the same family.

miR170 responds to drought stress and is expressed differently at different time points [39]. In response to salt stress, the expression of miR170 was down-regulated in cucumber grafted leaves and up-regulated in roots. miR170 regulates the expression of the *GRAS* transcription factor [45]. In our study, the expression of miR170-5p was significantly up-regulated after cucumber seedlings were exposed to high-temperature stress and Spd.

miR5077 was found conserved in *Bouteloua. gracilis* with high abundance [46]. This is in line with our findings. Dlo-miR5077 may be required for mature embryo formation [47]. In our study, miR5077 was down-regulated under high-temperature stress and up-regulated after exogenous Spd treatment, indicating that miR5077 may play an important role in the process of exogenous Spd increasing the tolerance of cucumber seedlings to high-temperature.

Of the other miRNAs responding to high-temperature and exogenous Spd, miR2275b-5p, miR479b, miR5222, and miR6475 have been rarely reported. miR2275b-5p, miR5222 and miR479b had similar expression pattern where their expression significantly down-regulated under high-temperature stress and sharply declined after exogenous Spd treatment. However, the expression of miR6475 was strongly induced by spraying Spd on cucumber seedlings under high-temperature stress. No target genes were predicted for miR2275b-5p or miR5222 in our study. Many genes targeted by miR479b, miR5077 and miR6475 were predicted, encoding proteins that had a wide range of functions and involved in a variety of metabolic pathways. That may imply that they were indeed reactive in response to high-temperatures and exogenous Spd.

### Genes and pathways involved in response to the improvement of cucumber high-temperature tolerance by exogenous spermidine

Since miRNAs are involved in the regulation of their target genes by degrading mRNA or inhibiting their translation [48], it is essential for understanding the regulation mechanism of miRNA-mediated Spd alleviation of damage to cucumber under high temperatures to identify the potential target genes. In our study, among the eight miRNAs that responded to high-temperature and exogenous Spd, five were predicted to target 107 genes. Functional annotations indicated that these genes encoded some proteins that were likely to be involved in response to high-temperature stress and Spd.

Many of these target genes were involved in the ubiquitin-proteasome pathway (UPP), including E3 ubiquitin ligase enzymes (F-box family protein, ring finger protein and U-box protein) and the 26S proteasome. E3 ubiquitin ligases play an important role in regulating the plant response to abiotic stress by regulating the abundance of critical stress-responsive transcription factors [49, 50]. In our study, miR6475 was down-regulated under high-temperature stress and up-regulated after exogenous Spd treatment. Therefore, its target gene, E3 ubiquitin protein ligase, may be up-regulated under high-temperature stress and down-regulated after treatment with Spd to relieve the damage caused by high-temperature stress through the UPP to degrade proteins that were misfolded or damaged under high-temperature stress. miR394a targeted an F-box family protein, which has substrate-recognition properties during ubiquitin-mediated proteolysis. Another study has shown that miR394 cleaves the F-box gene, whereas cleavage of the target mRNA is the dominant mode of regulation of plant miRNAs [45]. The F-box family gene which is also important in response to environment stress showed typical inverse expression trends compared to the expression of miR394a [51, 52]. In summary, exogenous Spd may alleviate the injury to cucumber seedlings under high-temperature through the UPP.

It has been reported that spraying exogenous Spd on the leaves of cucumber seedlings could increase the activity of antioxidant enzymes and promote the activity of the ascorbic acid-glutathione circulatory system. In our study, many genes encoding related proteins were identified, including superoxide dismutase, peroxidase, anti-cyclic-acid transporter (targeted by miR6475), and sulfite oxidase (targeted by miR479b), among others. It was determined that miR479b and miR6475 may participate in the response to exogenous Spd under high-temperature stress by regulating antioxidant system-related proteins, reducing the degree of membrane lipid peroxidation caused by high-temperature stress and removing reactive oxygen species to alleviate the high-temperature damage.

Surprisingly, target genes of miR6475 are involved in a wide range of regulatory pathways. miR6475 targets two transcription factors, myb domain proteins and no apical meristem domain transcription factor, which have been reported to activate stress-related genes. In addition to the above functions, it also targets proteins that respond to abiotic stress, ferredoxin associated with photosynthesis, and a series of protein kinases, among others. GO annotation and KEGG pathways analysis showed that miR6475 and its target genes are involved in a variety of biological functions and a number of significantly enriched metabolic pathways. Therefore, further study is necessary to explore the role of miR6475 in the regulation of exogenous Spd alleviating high-temperature stress injury.

In addition, eight target genes encoded unknown proteins. For some miRNAs (miR170-5p, miR2275b-3p, and miR5222), no target genes were identified. This may be because not all miRNAs regulate their target genes by cleavage mechanisms but may silence their target genes by inhibiting translation. In addition, different temporal or spatial expression of a miRNA and its target gene may lead to insufficient target gene cleavage.

The miRNA responsive network associated with the improvement of the high-temperature tolerance of cucumber by Spd is complex, including signalling pathways, the activation of genes responding to Spd and high-temperature stress, and the synthesis of various regulatory proteins. In this study, we identified some pathways that might be involved in the Spd-mediated improvement of cucumber high-temperature tolerance, such as glutathione metabolism (ko00480), ribosome biogenesis in eukaryotes (ko03008), and plant hormone signal transduction (ko04075). But only 5 pathways were significantly enriched. These pathways involved a wide range of functions, including RNA metabolism, protein synthesis, processing and degradation, and the biosynthesis of secondary metabolites.

## Conclusions

In summary, our study identified the first set of miRNAs that responded to both high-temperature and exogenous Spd in cucumber seedlings. The identification of these miRNAs and their target genes will help us to understand the molecular mechanism by which Spd alleviates high-temperature stress in cucumber seedlings, which provides useful information about miRNAs participating in the mechanism regulating the Spd-mediated improvement of cucumber high-temperature tolerance and has important significance for guiding cucumber high-temperature tolerance breeding.

## Methods

### Plant materials and treatments

The cucumber cultivar ‘Jinchun No. 2’, which was purchased from the Tianjin Kernel Cucumber Research Institute (Tianjin, China), has been shown to be a high temperature-sensitive cucumber cultivar (*Cucumis sativus* L.). A stock solution of Spd (1GM, Sigma, USA) was prepared and stored at 4 °C. The stock solution was diluted to 1.0 mmol·L^− 1^ for experimental use.

The experiment was performed in the automatic control glasshouse of the Pailou experimental base of Nanjing Agricultural University (118°46′E, 32°03′N) from October 2015 to November 2016. To ensure the germination of cucumber seeds, the full seeds were soaked in 55 °C water for 4 h and then placed in a constant temperature oscillator (HZ-8811 K, Taicang, China) under moist and dark conditions at 28 ± 1 °C for 24 h. The germinated seeds were sown in plastic pots (calibre × ground diameter × height = 10 cm × 7 cm × 8 cm, one seed per pot) filled with an organic substrate (vinegar residue: peat: vermiculite = 2:2:1 (by volume), Peilei, Zhenjiang, China), and transferred to the greenhouse. The seedlings were watered well to maintain the humidity at approximately 60%. In the greenhouse, the plants were grown in natural light, and the average daily photosynthetic photon flux density (PPFD) was 550 μmol·m^− 2^·s^− 1^. The temperature was 25~ 29 °C during the day and 15~ 19 °C at night. The relative air humidity was 60%~ 70%. After the complete development of the second leaf, healthy individuals grown at the same time were selected to be transferred to two identical artificial growth chambers (RXZ-500D, Ningbo, China) in which the day/night temperature was set to 28 ± 1 °C/18 ± 1 °C, the photoperiod was 12 h, the PPFD was 350 μmol·m^− 2^·s^− 1^, and the relative air humidity was 70~ 75%.

After 2 d of pre-cultivation, one chamber’s temperature was set to 42 ± 1 °C/32 ± 1 °C, and the other was maintained at 28 ± 1 °C/18 ± 1 °C. The seedlings were watered well to maintain the humidity at approximately 60%. During the treatments, every evening at 5 o’clock, half of the seedlings in both chambers were sprayed evenly on both sides of the leaves with 1.0 mmol·L^− 1^ Spd from a plastic spray bottle (N-5339, Shanghai, China). The other half of the seedlings was sprayed with the same amount of deionized water as a control. This procedure was performed for 7 d in a row. The experiment consisted of four treatments: (1) CW: seedlings were sprayed with deionized water and maintained at 28 ± 1 °C/18 ± 1 °C; (2) CS: seedlings were sprayed with 1.0 mmol·L^− 1^ Spd and maintained at 28 ± 1 °C/18 ± 1 °C; (3) HW: seedlings were sprayed with deionized water and maintained at 42 ± 1 °C/32 ± 1 °C; (4) HS: seedlings were sprayed with 1.0 mmol·L^− 1^ Spd and maintained at 42 ± 1 °C/32 ± 1 °C. After 7 d, the growth index of the cucumber seedlings was analysed. The clean fully expanded third leaves of eight seedlings randomly selected from each treatment were cut into pieces after removed the venation and margin, then they were mixed evenly and sampled. The samples were frozen in liquid nitrogen and stored at − 80 °C immediately.

### Determination of growth index

Eight seedlings were randomly selected from each treatment group to measure the growth index of cucumber seedlings. The plant height (distance from the cotyledon to the growing point) was measured with a ruler, and the stem diameter (cotyledonary node position) was measured with a Vernier calliper. These seedlings were divided into shoots and roots, rinsed with deionized water and blotted dry. The fresh weight of shoots was determined. The EPSON EXPRESSION 1680 leaf area / root volume scanner (Epson America Incorporation, California, United States) and WinRHIZO image analysis software (Regent Instruments Incorporation, Quebec, Canada) were used to measure the leaf area of the third functional leaves from the base of the cucumber seedlings. The shoots were ground at 115 °C for 15 min and baked at 75 °C until they maintained a constant weight. Then, the dry weight of these shoots was determined.

### Small RNA libraries construction and sequencing

Total RNA was extracted from three replicates of each treatment with Trizol reagent (Invitrogen, Carlsbad, CA) [39], and each treated sample was obtained by homogeneously mixing the completely expanded third leaves of eight seedlings. High-purity (OD260/280 between 1.8 and 2.2) and high-integrity (RNA integrity number, RIN ≥7.5) RNA samples from the CW, CS, HW and HS groups were selected to construct sRNA libraries (CW, CS, HW and HS). Then, high-throughput sequencing was performed on a HiSeq 2000 instrument (Illumina, USA). The data were processed by the following steps: (1) Total RNA was extracted from samples, and PAGE was used to separate RNA segments of different size. The band containing RNA sequences of 18–30 nt was excised from the gel, and the small RNAs were recovered. (2) Next, 3′ adaptors were added to the small RNAs, and the samples were mixed and centrifuged. The adaptors were ligated to the small RNAs based on a suitable temperature in a period of time. (3) Then, 5′ adaptors were added, and the samples were mixed and centrifuged. The adaptors were ligated based on a suitable temperature in a period of time. (4) On a PCR machine, the adaptor-ligated products were reverse-transcribed into double-stranded sequences, and these double-stranded sequences were then PCR-amplified according to certain procedures. (5) PAGE was used to separate and purify the PCR products. The purified products were resuspended in EB solution. (6) The Agilent 2100 Bioanalyzer and ABI Step One Plus Real-Time PCR System were used to determine the quality and yield of the RNA libraries.

### Raw data pre-processing

The basic figure from sequencing was converted into sequence data by the base calling step. Such sequence data called ‘raw reads’ was stored in a fastq format. Before doing any further analysis, quality control was required in order to detect whether the data was qualified. In addition, filtering of raw data was needed to decrease data noise. In a fastq format file, one read was represented by four lines: The first and third lines were names of this read, generated by sequencing machine. The second line was the sequence. The fourth line represented the sequencing quality [53] of this read. Each character in this line showed the sequencing quality of the base on the same position in the second line. The actual quality was the corresponding ASCII value of the letter minus 64. The quality of HiSeq sequencing ranges from 0 to 41. This quality will be used in the criteria for filtering out low quality reads. The filtered data was used to draw figures of base composition and base quality for clean data to visually check the sequencing quality. To obtain the final clean reads, we evaluated the raw reads by filtering out the contaminant reads and made an initial judgment on the data. The raw reads was processed by the following steps: (1) The low quality reads, with at least 4 base whose quality less than 10, was removed. (2) The reads which proportion of N were greater than 10 was removed. ‘N’ indicated that base information cannot be determined. (3) The reads with 5′ primer contaminants was removed. (4) The reads without the insert was removed. (5) The reads without 3′ primer was removed. (6) The reads with poly A was removed. (7) The reads shorter than 18 nt was removed [33, 54]. After the above processing, the clean reads was obtained. The same sequences were grouped together which were called ‘unique reads’. The ‘total reads’ indicated the number of sequences. Then, the unique reads and total reads were counted. In addition, the length distribution of the clean reads in the CW, CS, HW and HS libraries was calculated.

### Classification and annotation of small RNAs

The clean reads were mapped to the Cucumber Genomics Database (http://www.icugi.org/cgi-bin/ICuGI/genome/home.cgi?ver=2&organism=cucumber) using SOAP to analyse the expression and distribution of sRNAs, and no mismatch was allowed [55]. The perfectly matched sequences were mapped to the GenBank (ftp://ftp.ncbi.nlm.nih.gov/genbank/) and Rfam (http://rfam.janelia.org/) databases using BLASTN to annotate rRNA, snoRNA, snRNA and tRNA sequences. The sequences that matched the repeat sequences were removed. In addition, the sequences that matched the exon and intron sequences that represented mRNA degradation fragments were also removed. Then, the sRNAs were matched to the miRBase 21.0 database (http://www.mirbase.org/ftp.shtml) using BLAST to identify the known cucumber miRNAs. A perfect match was required between the sRNAs and the precursor sequences. However, the reads aligning to the mature miRNA in miRBase with at least 16 nt overlap were allowed offsets. The miRNAs that satisfied both of the above criteria were counted to determine the expression of the identified miRNAs and analysed to determine the base bias at the first position of identified miRNAs with a certain length and the base bias at each position of all identified miRNAs. Finally, all alignments and annotations were summarized.

The remaining sequences (called ‘unannotated sequences’) which mapped to antisense exons, introns or intergenic regions of the genome and did not map to any other RNA were used to predict novel miRNAs by exploring potential miRNA precursor sequences with characteristic hairpin structures. There are many ways to predict novel miRNAs, but no method is clearly superior [56]. In our study, we used the Mireap software (http://sourceforge.net/projects/mireap/) which is suitable for plants to predict novel miRNA by exploring the secondary structure, the Dicer cleavage site and the minimum folding free energy (MFE) of the unannotated small RNA reads which could be mapped to genome [57]. And this method has been widely used in many recent studies [58, 59]. The prediction of novel miRNA candidates included the base bias on the first position among small RNA candidates with a certain length and on each position among all small RNA candidates. Based on this summary, the prediction accuracy could be assessed according to the base bias of known miRNAs.

### Screening of miRNAs responsive to high-temperature stress and exogenous spermidine

The expression levels of known miRNAs and novel miRNAs in the four libraries were calculated and normalized as transcripts per million according to the formula: normalised expression = actual miRNA count / total count of clean reads × 1,000,000. The expression of miRNAs with an abundance of zero was modified to 0.01 for further analysis [60]. Then, the normalized results were used to calculate the fold-change and *p*-value. In order to avoid errors, miRNAs only expressed in one library were removed and did not participate in differential expression analysis.

We analysed the expression of the miRNAs in the four libraries. Then, the libraries were compared pair-wise to find the differentially expressed miRNAs. We divided them into four comparison pairs: (1) CS/CW: differential expression analysis of miRNAs between the CS and CW libraries, where CW was the control; (2) HW/CW: differential expression analysis of miRNAs between the HW and CW libraries, where CW was the control; (3) HS/CW: differential expression analysis of miRNAs between the HS and CW libraries, where CW was the control; and (4) HS/HW: differential expression analysis of miRNAs between the HS and HW libraries, where HW was the control. The differential expression of miRNAs was calculated by the following formula: fold-change = log_2_ (CS/CW, HW/CW, HS/CW or HS/HW). A miRNA was considered to be differentially expressed between the two compared libraries in each comparison pair when | Fold-change | = | (log_2_ (CS/CW, HW/CW, HS/CW or HS/HW) | > 1 and *p*-value < 0.05. The *p*-value was calculated according to previous method [61]. For example, A fold-change = log_2_ (CS/CW) > 1 indicated that the miRNA expression in CS library was significantly increased when compared with CW library; and a fold-change < − 1 indicated that the miRNA expression was significantly down-regulated.

Four sets of results from the four comparison pairs were obtained by the differential analysis, where miRNAs differentially expressed in the CS/CW comparison pair may be related to the response to exogenous Spd, miRNAs differentially expressed in the HW/CW comparison pair may be related to the response to high-temperature stress, miRNAs differentially expressed in the HS/CW comparison pair may be related to the interaction of high-temperature stress and exogenous Spd, and miRNAs differentially expressed in the HS/HW comparison pair may be related to the response to exogenous Spd under high-temperature stress.

The common and specific miRNAs that were differentially expressed in the CS/CW, HW/CW, HS/CW and HS/HW comparison pairs were then screened by Venn diagrams. Among them, the commonly differentially expressed miRNAs in the HW/CW and HS/CW comparison pairs may be related to high-temperature stress. The commonly differentially expressed miRNAs in the CS/CW, HS/CW, and HS/HW comparison pairs may be associated with exogenous Spd. However, the focus of this study was to explore miRNAs that may respond to both high-temperature stress and exogenous Spd, who may play important roles in the regulation of exogenous Spd-mediated improvement of high-temperature tolerance in cucumber seedlings. Thus, differentially expressed miRNAs that were common to all the four comparison pairs (CS/CW, HW/CW, HS/CW and HS/HW) were selected.

### Prediction and analysis of the genes targeted by high-temperature stress-responsive and exogenous spermidine-responsive miRNAs

To identify target genes of the miRNAs responsive to high-temperature stress and exogenous Spd, we compared the miRNA sequences to the EST sequences of the cucumber genome. The bioinformatics software Target Finder [62] and psRobot [63] was used to predict genes targeted by miRNAs involved in the response to both high temperature and exogenous Spd, taking the intersection as the predicted result. The intersections were the same genes targeted by the same miRNAs [64, 65]. The alignment of each miRNA with their putative targets was required to meet the criteria of a previous study [60].

To understand the biological functions of the target genes, GO annotation and KEGG were used to screen the biological functions and the main biochemical and metabolic pathways involved in the candidate genes. This method first maps all target gene candidates to GO terms in the database (http://www.geneontology.org/). Then, the number of genes successfully mapped to each term was counted. They were classified into three categories (biological process, cellular component and molecular function). The number of genes from the three categories were used to infer the function of the miRNAs. Next, a hypergeometric test was used to find significantly enriched GO terms in the target gene candidates compared to the reference gene background. GO terms with a corrected *p*-value < 0.05 were defined as significantly enriched in target gene candidates.

Similarly, the target genes were also assigned to KEGG terms, and the KEGG metabolic pathways (corrected *p*-value < 0.05) that were significantly enriched in the candidate target genes were determined by a hypergeometric test, and the pathogenic enrichment analysis was able to identify the candidate genes involved in the most important biochemical metabolic pathways and signal transduction pathways.

### Verification of miRNA responding to high temperature and exogenous spermidine and their potential target genes by qRT-PCR

Five differentially expressed known miRNAs, six differentially expressed novel miRNAs and six target genes that responded to exogenous Spd and/or high-temperature stress were randomly selected to verify the reliability of the high-throughput sequencing by qRT-PCR.

The main steps for the verification of miRNAs were performed as follows: Small RNAs were extracted from the leaves of plants in the CW, CS, HW and HS groups using the miRcute miRNA isolation kit (DP501, Tiangen, China). The small RNAs were then reverse transcribed to cDNA using the miRcute miRNA first-strand cDNA synthesis kit (kr201, Tiangen, China). Finally, qRT-PCR was performed using the miRcute miRNA qPCR Detection kit (SYBR Green) (FP401, Tiangen, China) in a Step One ™ Real-time PCR System (Applied Biosystems, Singapore). The miRNA forward primers were designed based on the miRNAs sequences. The universal reverse primers can be found in the Super Real Fluorescence Quantification Kit (SYBR Green) (FP205, Tiangen, China). The U6 snRNA was used as an internal reference for normalization [39].

The main steps for the validation of target genes were performed as follows: Total RNAs were extracted from the leaves of four treated samples using the RNA simple Total RNA kit (DP419, Tiangen, China). Then, the total RNAs were reverse transcribed to cDNA using the Fast Quant RT-Kit (kr106, Tiangen, China). Finally, real-time PCR was performed on the Step One ™ Real-time PCR System (Applied Biosystems, Singapore) using the Super Real Fluorescence Quantification Kit (SYBR Green) (FP205, Tiangen, China). The target gene primers were designed by Beacon Designer 7.9. The cucumber actin gene was used as an internal reference to normalize the qRT-PCR data.

All reactions were carried out according to the manufacturer’s instructions and repeated three times. Additional file 9: Table S8 lists the sequences of the primers used in these experiments.

The data were statistically analysed using SAS Version 9.0 software (SAS Institute, NC, USA). Using Duncan’s multiple range test, the significance level was set at *p*-value *<* 0.05.

## Additional files

Additional file 1: **Figure S1.** Figures of base composition and base quality for sequencing quality control. (A) The base composition map of CW library; (B) The base quality map of CW library. (C) The base composition map of CS library; (D) The base quality map of CS library. (E) The base composition map of HW library; (F) The base quality map of HW library. (G) The base composition map of HS library; (H) The base quality map of HS library. The horizontal coordinates are position on reads, the vertical coordinates are percent of each base. (TIF 5475 kb)

Additional file 2: **Table S1.** Detailed information of known miRNAs identified from the CW, CS, HW and HS libraries. (XLSX 21 kb)

Additional file 3: **Table S2.** Detailed information of novel miRNA candidates identified from the CW, CS, HW and HS libraries. (XLSX 37 kb)

Additional file 4: **Table S3.** List of miRNAs significantly expressed differentially in the HW/CW and HS/CW comparison pairs. (XLSX 279 kb)

Additional file 5: **Table S4.** List of miRNAs significantly expressed differentially in the CS/CW, HS/CW and HS/HW comparison pairs. (XLSX 276 kb)

Additional file 6: **Table S5.** The interaction between high-temperature and Spd in miRNAs responsive to both high-temperature and Spd. (DOCX 15 kb)

Additional file 7: **Table S6.** Annotation of potential genes targeted by miRNAs responding to both high-temperature and exogenous spermidine. (DOCX 17 kb)

Additional file 8: **Table S7.** The KEGG metabolic pathways of gene candidates targeted by miRNAs responding to high-temperature and spermidine. (XLSX 21 kb)

Additional file 9: **Table S8.** Primers of miRNAs and target genes used for qRT-PCR. (DOCX 16 kb)

## Abbreviations

GO: Gene Ontology
KEGG: Kyoto Encyclopedia of Genes and Genome
MFE: Minimum folding free energy
miRNA: microRNA
nt: nucletide
PPFD: Photosynthetic photon flux density
snoRNA: small nucleolar RNA
snRNA: small nuclear RNA
Spd: Spermidine
sRNA: small RNA
UPP: Ubiquitin- proteasome pathway
