# Supplementary figures and images for: Identification of microRNAs associated with the exogenous spermidine-mediated improvement of high-temperature tolerance in cucumber seedlings (Cucumis sativus L.)
Source: BMC Genomics. 2018 Apr 24;19:285. doi: 10.1186/s12864-018-4678-x (PMC5937831; doi:10.1186/s12864-018-4678-x)

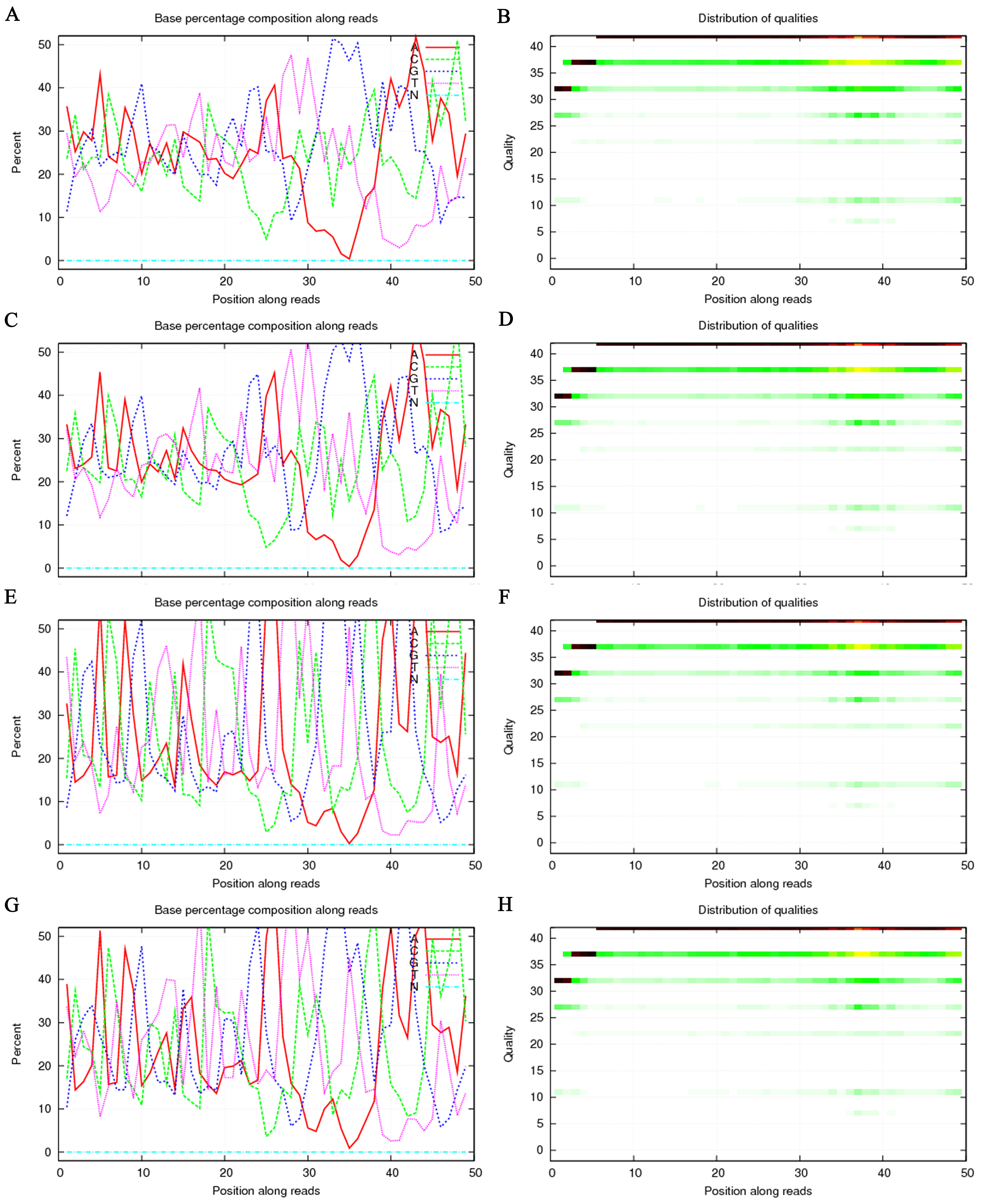

Supplement: Supplementary file 1 — Figure S1. Figures of base composition and base quality for sequencing quality control. (A) The base composition map of CW library; (B) The base quality map of CW library. (C) The base composition map of CS library; (D) The base quality map of CS library. (E) The base composition map of HW library; (F) The base quality map of HW library. (G) The base composition map of HS library; (H) The base quality map of HS library. The horizontal coordinates are position on reads, the vertical coordinates are percent of each base. (TIF 5475 kb) [file 12864_2018_4678_MOESM1_ESM.tif]
