# Supplementary material for: Identification of microRNAs associated with the exogenous spermidine-mediated improvement of high-temperature tolerance in cucumber seedlings (Cucumis sativus L.)
Source: BMC Genomics. 2018 Apr 24;19:285. doi: 10.1186/s12864-018-4678-x (PMC5937831; doi:10.1186/s12864-018-4678-x)
Supplement: Supplementary file 6 — Table S5. The interaction between high-temperature and Spd in miRNAs responsive to both high-temperature and Spd. (DOCX 15 kb) [file 12864_2018_4678_MOESM6_ESM.docx]

**Additional file 6: Table S5.** The interaction between high-temperature and Spd in miRNAs responsive to both high-temperature and Spd.

| **miRNA** | **Source** | **DF** | **Type III SS** | **M S** | **F-value** | **Pr > F** |
| --- | --- | --- | --- | --- | --- | --- |
| miR156d-3p | H | 1 | 3675.0000 | 3675.0000 | 24.7100 | 0.0011 |
|  | Spd | 1 | 671187.0000 | 671187.0000 | 4512.1800 | <.0001 |
|  | Spd*H | 1 | 3675.0000 | 3675.0000 | 24.7100 | 0.0011 |
| miR170-5p | H | 1 | 81729540.8000 | 81729540.8000 | 5944.8300 | <.0001 |
|  | Spd | 1 | 9660690.8000 | 9660690.8000 | 702.7000 | <.0001 |
|  | Spd*H | 1 | 109607940.8000 | 109607940.8000 | 7972.6500 | <.0001 |
| miR394a | H | 1 | 6.6901 | 6.6901 | 13.3800 | 0.0064 |
|  | Spd | 1 | 468.2501 | 468.2501 | 936.2500 | <.0001 |
|  | Spd*H | 1 | 6.8101 | 6.8101 | 13.6200 | 0.0061 |
| miR479 | H | 1 | 77763.0000 | 77763.0000 | 656.2300 | <.0001 |
|  | Spd | 1 | 129792.0000 | 129792.0000 | 1095.2900 | <.0001 |
|  | Spd*H | 1 | 1728.0000 | 1728.0000 | 14.5800 | 0.0051 |
| miR2275b-5p | H | 1 | 3.0301 | 3.0301 | 6.0500 | 0.0394 |
|  | Spd | 1 | 2182.4124 | 2182.4124 | 4354.0100 | <.0001 |
|  | Spd*H | 1 | 2.9701 | 2.9701 | 5.9300 | 0.0409 |
| miR5077 | H | 1 | 826875.0000 | 826875.0000 | 260.1500 | <.0001 |
|  | Spd | 1 | 384492.0000 | 384492.0000 | 120.9700 | <.0001 |
|  | Spd*H | 1 | 3115083.0000 | 3115083.0000 | 980.0500 | <.0001 |
| miR5222 | H | 1 | 2080.3333 | 2080.3333 | 891.5700 | <.0001 |
|  | Spd | 1 | 1776.3333 | 1776.3333 | 761.2900 | <.0001 |
|  | Spd*H | 1 | 40.3333 | 40.3333 | 17.2900 | 0.0032 |
| miR6475 | H | 1 | 4107.0000 | 4107.0000 | 966.3500 | <.0001 |
|  | Spd | 1 | 147.0000 | 147.0000 | 34.5900 | 0.0004 |
|  | Spd*H | 1 | 3675.0000 | 3675.0000 | 864.7100 | <.0001 |

DF: degree freedom, SS: Stdev Square, MS: Mean Square.

The greater the F-value (F-value>0.05) and the smaller the P-value (P-value<0.05), the more significant the difference.
