# Supplementary material for: Identification of microRNAs associated with the exogenous spermidine-mediated improvement of high-temperature tolerance in cucumber seedlings (Cucumis sativus L.)
Source: BMC Genomics. 2018 Apr 24;19:285. doi: 10.1186/s12864-018-4678-x (PMC5937831; doi:10.1186/s12864-018-4678-x)
Supplement: Supplementary file 7 — Table S6. Annotation of potential genes targeted by miRNAs responding to both high-temperature and exogenous spermidine. (DOCX 17 kb) [file 12864_2018_4678_MOESM7_ESM.docx]

**Additional file 7: Table S6.** Annotation of potential genes targeted by miRNAs responding to both high-temperature and exogenous spermidine.

| Family | miRNA  name | Number  of genes | Gene ID | Annotation of potential targets |
| --- | --- | --- | --- | --- |
| miR156 | miR156d-3p | 1 | Csa5M001030 | Putative uncharacterized protein P0704D04.7 |
| miR394 | miR394a | 3 | Csa3M133260 | leucine-rich repeat receptor-like protein kinase |
|  |  |  | Csa5M184300 | S-adenosyl-L-methionine-dependent methyltransferase |
|  |  |  | Csa6M087700 | F-box family protein |
| miR479 | miR479b | 2 | Csa2M406690 | Sulfite oxidase |
|  |  |  | Csa5M218740 | Cation/H(+) antiporter 15 |
| miR5077 | miR5077 | 5 | Csa1M073730 | Protein of unknown function DUF974 |
|  |  |  | Csa2M357280 | Cytochrome P450 |
|  |  |  | Csa3M017120 | cyclophilin type peptidyl-prolyl cis-trans isomerase |
|  |  |  | Csa4M007050 | 3-beta-hydroxysteroid-delta-isomerase |
|  |  |  | Csa7M049210 | ATP-dependent zinc metalloprotease FtsH |
| miR6475 | miR6475 | 96 | Csa1M002150 | adenylate kinase |
|  |  |  | Csa1M015790 | G patch domain-containing protein 1 |
|  |  |  | Csa1M050430 | Katanin p60 |
|  |  |  | Csa1M097670 | Shugoshin-1 |
|  |  |  | Csa1M163140 | Ribosomal protein |
|  |  |  | Csa1M533710 | E3 ubiquitin ligase |
|  |  |  | Csa1M571850 | Superoxide dismutase |
|  |  |  | Csa1M575100 | NAC domain protein |
|  |  |  | Csa1M589720 | serine-threonine protein kinase |
|  |  |  | Csa1M703040 | DNA helicase |
|  |  |  | Csa2M005950 | GDP-mannose transporter |
|  |  |  | Csa2M006170 | Nitrate transporter |
|  |  |  | Csa2M020910 | methanol dehydrogenase (Precursor) |
|  |  |  | Csa2M079640 | Translational activator GCN1 |
|  |  |  | Csa2M129150 | 3-ketoacyl-CoA thiolase |
|  |  |  | Csa2M170810 | Aspartyl/glutamyl-tRNA(Asn/Gln) amidotransferase subunit B |
|  |  |  | Csa2M223130 | stress-induced protein 1 |
|  |  |  | Csa2M297760 | Ethylene-responsive transcription factor |
|  |  |  | Csa2M351020 | U-box domain-containing protein |
|  |  |  | Csa2M379980 | Boron transporter |
|  |  |  | Csa2M401440 | Sucrose phosphate synthase |
|  |  |  | Csa2M403140 | RING finger protein 13 |
|  |  |  | Csa3M002670 | Rac-like GTP-binding protein |
|  |  |  | Csa3M002730 | 26S proteasome |
|  |  |  | Csa3M062610 | Mitochondrial import inner membrane translocase |
|  |  |  | Csa3M113400 | Ferric reductase oxidase |
|  |  |  | Csa3M124780 | Fructosamine kinase family protein |
|  |  |  | Csa3M128950 | pentatricopeptide repeat-containing protein |
|  |  |  | Csa3M153720 | Putative homeodomain-like transcription factor superfamily protein |
|  |  |  | Csa3M209460 | Harpin-induced protein |
|  |  |  | Csa3M222800 | Ferredoxin |
|  |  |  | Csa3M234000 | Fatty acid oxidation complex subunit alpha |
|  |  |  | Csa3M253500 | Acetyl-coenzyme A synthetase |
|  |  |  | Csa3M305660 | Chromodomain-helicase-DNA-binding protein 1-like |
|  |  |  | Csa3M576850 | Cyclic nucleotide-gated ion channel |
|  |  |  | Csa3M686720 | WGS project CAID00000000 data, contig chromosome 07 |
|  |  |  | Csa3M710220 | Glycosylphosphatidylinositol anchor biosynthesis protein 11 |
|  |  |  | Csa3M779010 | Harpin inducing protein 1-like 9 |
|  |  |  | Csa3M812210 | Protein N-terminal glutamine amidohydrolase |
|  |  |  | Csa3M820510 | Amino acid transporter family protein |
|  |  |  | Csa3M829270 | Peroxidase |
|  |  |  | Csa3M846050 | Gb\|AAD25600.1 |
|  |  |  | Csa3M848220 | Transcription factor |
|  |  |  | Csa4M010420 | homeodomain-like transcription factor superfamily protein |
|  |  |  | Csa4M123320 | Unknown protein |
|  |  |  | Csa4M166960 | 30S ribosomal protein S5 |
|  |  |  | Csa4M279830 | Oligoribonuclease |
|  |  |  | Csa4M280530 | RNA helicase |
|  |  |  | Csa4M337340 | Auxin efflux carrier family protein |
|  |  |  | Csa4M509540 | Patellin-3 |
|  |  |  | Csa4M646360 | Mitochondrial carrier protein |
|  |  |  | Csa4M658570 | Kelch-like protein |
|  |  |  | Csa5M139870 | ATPase family gene 2 protein |
|  |  |  | Csa5M148500 | GDSL lipase |
|  |  |  | Csa5M149860 | Putative uridine kinase |
|  |  |  | Csa5M153150 | Glutamate-cysteine ligase |
|  |  |  | Csa5M166980 | serine-threonine protein kinase |
|  |  |  | Csa5M172800 | MADS-box transcription factor 2 |
|  |  |  | Csa5M180300 | Dual specificity protein phosphatase |
|  |  |  | Csa5M217170 | Protein trichome birefringence-like 33 |
|  |  |  | Csa5M424880 | Similarity to DNA repair protein |
|  |  |  | Csa5M512940 | Serine/threonine-protein phosphatase |
|  |  |  | Csa5M523030 | Altered inheritance of mitochondria protein 32 |
|  |  |  | Csa5M524850 | L-aspartate oxidase |
|  |  |  | Csa5M527890 | clp protease |
|  |  |  | Csa5M599290 | AP-1 complex |
|  |  |  | Csa5M603280 | Nucleobase ascorbate transporter |
|  |  |  | Csa5M604410 | Myb-related protein-like |
|  |  |  | Csa5M623710 | Putative uncharacterized protein T14E10_70 |
|  |  |  | Csa5M634330 | HI0933 family protein (Precursor) |
|  |  |  | Csa5M637720 | Pectinesterase |
|  |  |  | Csa5M642130 | Pollen allergen Phl p 11 |
|  |  |  | Csa6M00090 | SWR1-complex protein 5 |
|  |  |  | Csa6M001760 | Cytochrome P450 |
|  |  |  | Csa6M128660 | Protein of unknown function DUF789 |
|  |  |  | Csa6M151130 | Stress responsive A/B barrel domain family protein |
|  |  |  | Csa6M176410 | Elongation factor 2 |
|  |  |  | Csa6M182120 | Pentatricopeptide repeat protein |
|  |  |  | Csa6M403620 | Domain of unknown function DUF1338 |
|  |  |  | Csa6M410680 | Outer envelope pore protein 37, chloroplastic |
|  |  |  | Csa6M431760 | E3 ubiquitin-protein ligase ICP0 |
|  |  |  | Csa6M486840 | Cyclin-dependent protein kinase-like |
|  |  |  | Csa6M497170 | cDNA, clone: J065213I19, full insert sequence |
|  |  |  | Csa6M502010 | Unknown protein |
|  |  |  | Csa6M525360 | Multidrug resistance protein ABC transporter family |
|  |  |  | Csa7M033370 | Nuclear protein DGCR14 |
|  |  |  | Csa7M212680 | UPF0505 protein C16orf62-like |
|  |  |  | Csa7M214190 | Cell differentiation protein rcd1 |
|  |  |  | Csa7M236800 | Callose synthase 7 |
|  |  |  | Csa7M237890 | Putative receptor-like protein kinase |
|  |  |  | Csa7M325180 | Protein FAM188A |
|  |  |  | Csa7M325200 | ATP-dependent RNA helicase |
|  |  |  | Csa7M330380 | Unknown protein |
|  |  |  | Csa7M330400 | CLIP-associating protein |
|  |  |  | Csa7M433170 | Polygalacturonase |
|  |  |  | CsaUNM013090 | Subtilisin-like protease |
