# Supplementary material for: Identification of microRNAs associated with the exogenous spermidine-mediated improvement of high-temperature tolerance in cucumber seedlings (Cucumis sativus L.)
Source: BMC Genomics. 2018 Apr 24;19:285. doi: 10.1186/s12864-018-4678-x (PMC5937831; doi:10.1186/s12864-018-4678-x)
Supplement: Supplementary file 9 — Table S8. Primers of miRNAs and target genes used for qRT-PCR. (DOCX 16 kb) [file 12864_2018_4678_MOESM9_ESM.docx]

**Additional file 9: Table S8.** Primers of miRNAs and target genes used for qRT-PCR.

| miRNAs and targets | Forward primers (5'-3') | Reverse primers (5'-3') |
| --- | --- | --- |
| U6 snRNA | GGGGACATCCGATAAAATT | TGTGCGTGTCATCCTTGC |
| miR394a | TTGGCATTCTGTCCACCTCC |  |
| miR408 | TGCACTGCCTCTTCCCTGGCTG |  |
| miR408-3p | ATGCACTGCCTCTTCCCTGGC |  |
| miR5077 | ATTCACGTCGGGTTCACCA |  |
| miR6475 | TCTTGAAAGTAAAGGAGC |  |
| novel-mir37 | TTCAGGTTGGGTTGGATTGAACC |  |
| novel-mir79 | GGAATGTTGTCTGGCTCGAGG |  |
| novel-mir138 | GAGAATTAAATGACTGTGGGCTT |  |
| novel-mir179 | CAACCGAGACTAAATTCTAAC |  |
| novel-mir204 | CAAGAATCTAGAAATAACGGACT |  |
| novel-mir265 | CCATCAGAGGATATGGAGAACT |  |
| actin | CAGGAATCCACGAAACTACT | AGACCCTCCAATCCAAACAC |
| Csa5M184300 | CAATACCCACCCTTTCTT | CACTGAGACAATGGAACT |
| Csa5M585970 | GAACTGGTGGAGATGAAG | TCTGATTCTGTTATGTCTACC |
| Csa3M215590 | TTCACCACATCATTCCAA | TCACCCATTCTTCTTCTAAA |
| Csa3M017120 | ATCTCGTTTGACTCTTCC | CATCGCCATCTCTAATCA |
| Csa6M00090 | AGAGGACTTGGATGCTTA | CGTTCAAACTCCCTGTAA |
| Csa1M163140 | TGAGAATGAACTGGAAGAG | CCCTGAGAACTTGATGAA |
